# Effect of Coal Tar Components and Thermal Polycondensation Conditions on the Formation of Mesophase Pitch

**DOI:** 10.3390/ma18051002

**Published:** 2025-02-24

**Authors:** Lei Zhang, Haocheng Zhao, Lei Zhang, Ruikang Song, Qi Wang, Ziqing Liu

**Affiliations:** 1 College of Geology and Environment, Xi’an University of Science and Technology, Xi’an 710054, China; haocheng202302@163.com (H.Z.); srk781388595@163.com (R.S.); 18391871932@163.com (Q.W.); ziqing200112@163.com (Z.L.); 2 Key Laboratory of Coal Resources Exploration and Comprehensive Utilization, Ministry of Natural Resources, Xi’an 710021, China; 3 China National Heavy Machinery Research Institute Co., Ltd., Xi’an 710032, China; leizh666@sohu.com

**Keywords:** thermal polymerization, reaction conditions, product characterization, analytical testing

## Abstract

This study focuses on the preparation of mesophase pitch via the thermal polycondensation of heavy components from low-temperature coal tar. By altering the coal tar composition through distillation, we investigated the impact of various coal tar components and reaction conditions on the properties of the resulting mesophase pitch. Techniques such as infrared spectroscopy, nuclear magnetic resonance, optical structure analysis, and family-component analysis were employed to analyze both the coal tar and mesophase pitch. The primary objective was to provide a comprehensive understanding of mesophase pitch preparation and the underlying transformation mechanisms of coal tar at the molecular, chemical, and functional group levels. Our findings revealed that mesophase pitch formation was driven by a combination of chemical reactions and physical processes. Increasing the distillation temperature reduced the number of alkyl substituents, shortened chain lengths, and promoted greater aromatic condensation. The optimal mesophase pitch content was achieved at a distillation temperature of 360 °C, a reaction temperature of 400 °C, and a holding time of 12 h, resulting in a predominantly inlaid structure. This work addresses a gap in the understanding of coal tar transformation, highlighting how the interplay between distillation temperature and reaction conditions affects the structural properties of mesophase pitch, with implications for improving its production and applications in carbon materials.

## 1. Introduction

The clean and efficient utilization of coal represents an inevitable tendency in the future development and transformation of the energy industry [1]. Given the current technical levels of coal pyrolysis and coal-tar upgrading, the processing capacity of tar is significantly lower than its production volume [2]. Coal tar exhibits characteristics such as high aromaticity, high carbon content, and ease of graphitization [3]. Improving the processing capacity of coal tar and developing new technologies can significantly enhance the comprehensive utilization of tar and reduce resource waste. Mesophase pitch is a high-purity pitch obtained through the pyrolysis of petroleum or coal tar, characterized by a unique layered structure that lies between the amorphous and crystalline states. This distinctive structure imparts excellent thermal stability, mechanical strength, and electrical conductivity, making it a crucial raw material for the production of high-performance carbon materials. In the manufacturing of carbon fibers and graphitized carbon, mesophase pitch undergoes high-temperature treatment, transforming into a layered graphite structure, thereby significantly enhancing the material’s strength and toughness. Furthermore, its applications are expanding in fields such as battery electrode materials, conductive coatings, and pyrolyzed carbonized materials. The unique properties of mesophase pitch play an essential role in various advanced industrial sectors, particularly in energy, electronics, and materials engineering [4,5,6]. Currently, advanced processes such as catalytic cracking, supercritical fluid technology, and molecular sieve adsorption offer more efficient methods for extracting mesophase pitch and optimizing the distillation of tar [7]. These technologies not only improve production efficiency but also enable precise control over product quality. They foster the advancement of the coal chemical industry towards higher added value and technological sophistication, meet the market demand for high-quality pitch materials, and contribute to environmental protection and energy conservation. The method we use to prepare mesophase pitch is the direct thermal polycondensation approach. This method is simpler in terms of the process compared to the aforementioned approaches while ensuring product quality: through the controlled thermal condensation of tar at elevated temperatures [8], it enhances the efficiency of the tar processing and results in the production of mesophase pitch with superior quality. By employing this approach, the processing time is reduced, and the yield of mesophase pitch is improved, contributing further to both economic and environmental benefits in the coal chemical industry [9,10]. Coal tar has the merits of having abundant resources, a low cost, high carbon content, good fluidity, and easy graphitization [11]. It is relatively easy to obtain a planar aromatic macromolecular structure through the thermal polymerization process. Consequently, coal tar is utilized as the precursor for the carbonaceous mesophase [12]. The formation of mesophase pitch is essentially a process of phase transformation, in which complex physical and chemical changes are constantly taking place, making it isotropic to anisotropic, a product of a certain degree of thermal decomposition and thermal condensation of the raw materials [13].

Mesophase pitches synthesized from various raw materials possess distinct structures and properties [14]. Aromatic molecules with more side chains or naphthenic structures are more likely to undergo thermal decomposition, aromatization, and thermal polycondensation to produce mesophase pitch at low temperatures. Liang [15] found that the heavier components have a greater reaction rate, which also increases with the molecular weight of the feedstock. Wang [16] found that the cyclic alkane structure can maintain the activity of the reaction system and facilitate the orderly growth of the intermediate phase [17].

The composition of the coal tar, the temperature, and the reaction time are important reaction conditions affecting the structure and properties of the carbonaceous mesophase during its preparation. The preparation of carbonaceous mesophases from carbon-rich feedstocks has been increasingly studied. But there are few reports that have systematically investigated the association between the various components of coal tar and the formation, structure, and properties of carbonaceous mesophases during thermal polycondensation. Based on the above literature, it can be deduced that the aromatic structure is the core component that constitutes the mesophase. The naphthenic structure provides an excellent reaction system, the content of QI directly affects the structure of the mesophase, and the components of the raw material have a great impact on the performance of the mesophase pitch. The preparation of ordered, high-quality mesophase pitch from inexpensive coal tar is a major research challenge. This study investigated the effect of coal tar composition on the structure and properties of the carbonaceous mesophase by altering the content of each component in the coal tar by distillation. Using the tar produced by coal pyrolysis as the target, the optimum reaction conditions were selected by controlling the temperature and time of the reaction to prepare a mesophase pitch with an ordered structure to investigate the effects of coal tar composition and reaction conditions on the orderliness of the mesophase and to elucidate the mechanism of the preparation of mesophase pitch from coal tar and the regulation of mesophase orderliness by additives [18,19]. This study uniquely elucidates the specific impact of coal tar components on the mesophase structure, bridging existing knowledge gaps in composition and reaction optimization for enhanced mesophase orderliness.

## 2. Experimental Procedures and Instrumental Analysis

### 2.1. Selection of Experimental Methods

Since Tayler’s discovery of optically anisotropic mesophase pitch in the 1960s, research on this material has been continuous. Various methods for preparing mesophase pitch have been explored, including direct thermal polymerization, catalytic thermal polymerization, and co-carbonization.

To reduce industrial application costs, we chose the simplest method, direct thermal polymerization, for our study. This method involves the carbonization of coal tar pitch, petroleum pitch, tar, or heavy oil under liquid-phase conditions at 350–560 °C, causing pyrolysis, aromatization, and polycondensation reactions during heating, ultimately yielding aromatic macromolecules with planar structures. Yang [20] investigated the transformation behaviors of mesophase in coal tar pitches with different softening points, using FT-IR and XRD to analyze the molecular structure of the pitches. They found that high aromaticity and molecularly layered structures facilitate carbon microsphere growth and mesophase formation. Additionally, Yang [21] performed further heat treatment on pyrolysis fuel oil-based pitch, confirming the generation of carbon microspheres at temperatures below 400 °C. Above 400 °C, the spherical mesophase structures grew larger. Li [22] used high-pressure thermal treatment of naphthenic vacuum gas oil fractions with different aromatic contents and analyzed the influence of raw material structures on mesophase pitch formation. The results indicated that raw materials with higher aromaticity, more naphthenic structures, and fewer alkyl side chains were more likely to form mesophase pitches with a wide optical structure, lower softening points, and more ordered crystalline structures.

### 2.2. Experimental Details

**Figure 1:**
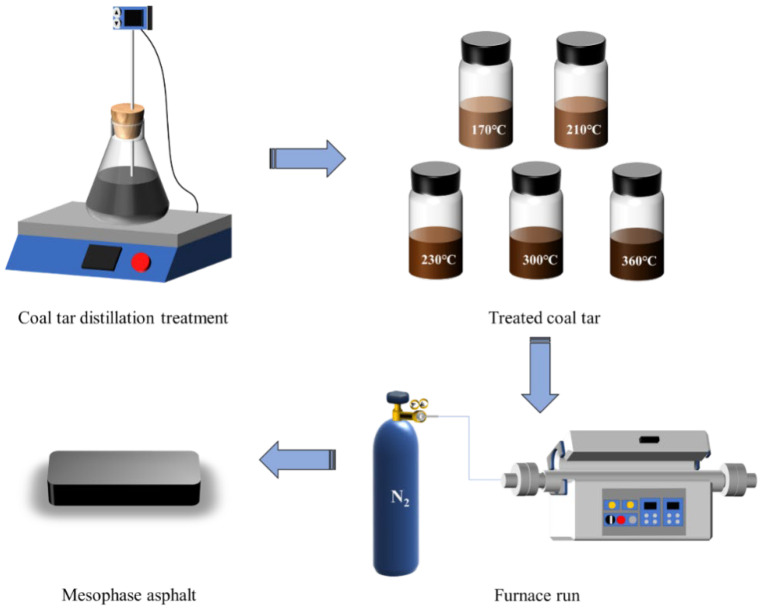
Experimental process.

Based on the thermal processing characteristics of coal tar, an appropriate temperature was selected, and experiments were conducted accordingly. The experimental process is shown in Figure 1. The raw coal tar was placed in the distillation equipment (magnetic stirrer with heating plate) with the setting temperatures of 170 °C, 210 °C, 230 °C, 300 °C, and 360 °C [6,23]. After reaching the setting temperature, the coal tar was held for 6 h. The coal tar obtained at different distillation temperatures was then characterized. A mass of 10 g of distillate-treated coal tar was weighed and placed in the isothermal zone of the high-temperature, high-pressure tubular furnace, the reactor lid was sealed, and the reaction kettle was rinsed repeatedly with high-purity nitrogen. The furnace was then pressurized to 1 MPa and left for 20 min. The temperature was increased to the final temperature (380 °C, 400 °C, 420 °C) at a rate of 2 °C/min and held for a certain time (3 h, 6 h, 9 h, 12 h, 15 h), then cooled to room temperature and the sample was retrieved and characterized.

### 2.3. Sample Characterization

#### 2.3.1. Component Analysis

A mass of 2.5 g of the sample was mixed with 75 mL of n-heptane under ultrasonic treatment. The mixture was then vacuum-filtered to separate the n-heptane-soluble fraction (HS) and the n-heptane-insoluble fraction (HI). The HI fraction was dried and weighed, then mixed with 75 mL of toluene under ultrasonic treatment. Vacuum filtration was performed again to separate the n-heptane-insoluble/toluene-soluble fraction (HI-HS) and the toluene-insoluble fraction (HI). The toluene-insoluble fraction (HI) was then dried and weighed, followed by mixing with 75 mL of quinoline under ultrasonic treatment. After vacuum filtration, the toluene-insoluble/quinoline-soluble fraction (TI-QS) and quinoline-insoluble fraction (QI) were separated.

#### 2.3.2. ^1^H-NMR Analysis

Nuclear magnetic resonance (NMR) spectra were recorded using a Bruker 400 M instrument (Purchased from Karlsruhe, Germany). A known amount of the sample was dissolved in deuterated chloroform. Structural calculations were performed using the B-L method. Based on chemical shifts, the hydrogen distribution was categorized into four regions: aromatic protons (6.5–9.5 ppm), α-protons of the aromatic side chain (2.0–4.5 ppm), β-protons of the aromatic side chain (1.0–2.0 ppm), and γ-protons of the aromatic side chain (0.0–1.0 ppm).

#### 2.3.3. Optical Structural Analysis

The asphalt produced from the thermal conversion reaction was placed into a mold, followed by injection of a cold embedding agent. After curing, the sample was demolded, rough-ground, and polished. Finally, the anisotropic mesophase morphology was observed under a Leica DM750P optical microscope (Purchased from Wetzlar, Germany).

#### 2.3.4. FT-IR Analysis

Fourier transform infrared (FT-IR) analysis was conducted using a Bruker VERTEX 70 spectrometer (Purchased from Karlsruhe, Germany). A suitable amount of the sample was prepared using the KBr pellet method for infrared spectroscopy. The spectral range covered was from 4000 to 400 cm^−1^, with 28 scans and a resolution of 0.4 cm^−1^.

The aromaticity index (I_ar_) quantifies the aromatic nature of the material, while the CH₃/CH₂ branching index serves as an indicator of the alkyl side chain density. The calculation formulas are as follows [24]:

(1) $${\mathrm{CH}}_{3}/{\mathrm{CH}}_{2}=\frac{\mathrm{Abs}\cdot 2950\;{\mathrm{cm}}^{-1}}{\mathrm{Abs}\cdot 2920\;{\mathrm{cm}}^{-1}}$$

(2) $$I_{\mathrm{ar}}=\frac{\mathrm{Abs}\cdot 3050\;{\mathrm{cm}}^{-1}}{\mathrm{Abs}\cdot 3050\;{\mathrm{cm}}^{-1}+\mathrm{Abs}\cdot 2920\;{\mathrm{cm}}^{-1}}$$

where

Abs·2950 cm^−1^ corresponds to the absorption peak area of the methyl group;
Abs·2920 cm^−1^ corresponds to the absorption peak area of the methylene group;
Abs·3050 cm^−1^ corresponds to the absorption peak area of the aromatic hydrogen.

#### 2.3.5. Gas Chromatography Analysis

Gas chromatography (GC) analysis of the coal tar was performed using a GC1100 chromatograph (Beijing Purui Analytical Instrument Co., Ltd., Beijing, China). The quality of the tar was evaluated using the gas chromatography simulated distillation method. The principle of this technique involves the use of a non-polar chromatographic column with a high degree of separation. Under a linear temperature-programmed condition, the retention times of known mixtures are first determined. Subsequently, the sample components are separated in sequence based on their boiling points under the same chromatographic conditions, allowing for the determination of the percentage composition of each component.

## 3. Results and Discussion

### 3.1. Coal Tar Composition Analysis

#### 3.1.1. Physical Properties

The coal tars obtained after distillation treatments at 170 °C, 210 °C, 230 °C, 300 °C, and 360 °C are recorded as CT_1_, CT_2_, CT_3_, CT_4_, and CT_5_, respectively. The raw coal tar is recorded as CT_0_. The group composition and elemental analysis of raw coal tar and distillation-treated coal tar are shown in Table 1. HS decreased with increasing distillation temperature, HI-TS and TI-QS increased with increasing distillation temperature. The value of QI was small and negligible. HS was mainly composed of molecules with two to three rings. With increasing distillation temperature, such small molecules were separated by distillation [25]. Consequently, the content of HS demonstrated a significant variation with temperature.

**Figure 2:**
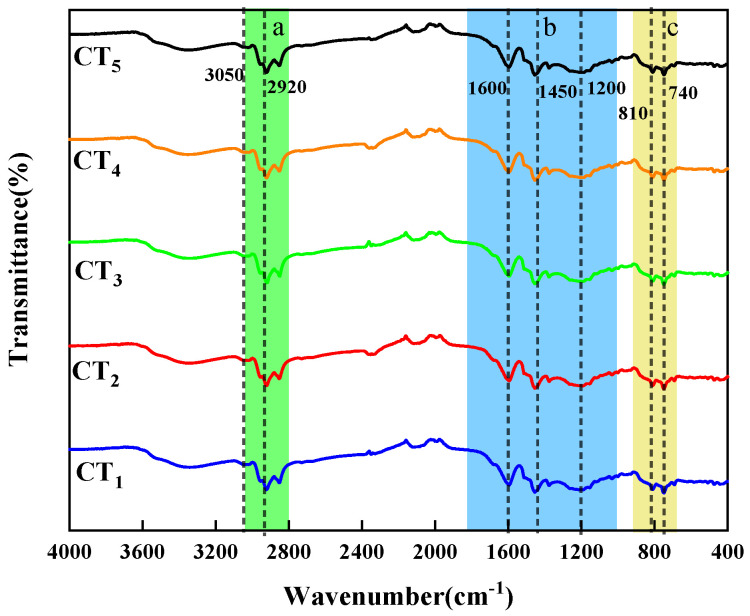
Infrared spectrum of distilled coal tar (a: 2800~3000 cm^−1^, b: 1000~1800 cm^−1^, c: 700~900 cm^−1^).

Figure 2 shows the infrared spectrum of distilled coal tar. The coal tar has large absorption peaks at 3050 cm^−1^ and 1600 cm^−1^, which are the vibrational absorption peaks of C-H on the benzene ring and C=C on the aromatic ring, indicating that coal tar has a thick-ringed aromatic and benzene ring-like structure. There is a clear absorption peak at 2920 cm^−1^ for the C-H vibrational absorption peak of saturated methylene and a C-H vibrational absorption peak of methylene near 1450 cm^−1^. This indicates that coal tar is rich in alkyl side chains and alkane structures. As the distillation temperature increases, the content of the lighter components decreases. The characteristic peak signals of the alkyl side chain and alkane structures of coal tar become weaker. There is a large absorption peak near 3430 cm^−1^, which is a stretching vibration peak of -OH, probably produced by phenol-like substances. Symmetric and asymmetric vibrational absorption peaks in the C-H plane at 2920 cm^−1^, 2853 cm^−1^, and 1375 cm^−1^ indicate the presence of the -CH_2_-CH_3_ functional group in the coal tar. The presence of an absorption peak at 750 cm^−1^ but not at 720 cm^−1^ indicates the absence of longer alkyl side chains in the coal tar [26].

**Figure 3:**
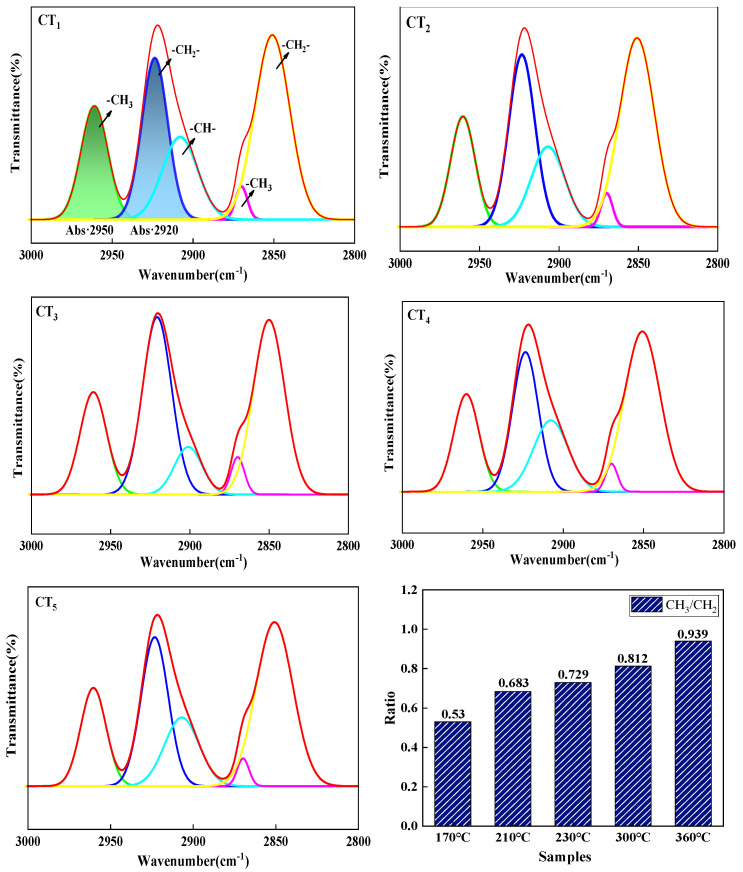
Peak fitting pattern of coal tar at 2800~3000 cm^−1^.

In order to determine the structure of the coal tar components more accurately, the infrared spectrum was divided into three regions, a, b and c: a represents 2800~3000 cm^−1^, b represents 1000~1800 cm^−1^, and c represents 700~900 cm^−1^. The three regions were subjected to split-peak fitting. Figure 3 shows the peak splitting fits for coal tar at 2800 to 3000 cm^−1^. This region consists mainly of five functional groups: 2960 cm^−1^ and 2923 cm^−1^ are the telescopic vibrational peaks of asymmetric -CH_3_ and -CH_2_-; 2870 cm^−1^ and 2850 cm^−1^ are the telescopic vibrational peaks of symmetric -CH_3_ and -CH_2_-; and 2907 cm^−1^ is the telescopic vibrational peak of -CH-. CH_3_/CH_2_ indicates the abundance of alkyl side chains. The smaller the value of CH_3_/CH_2_, the greater the number of alkyl substituents and the longer the chain length. The values of CH_3_/CH_2_ for CT_1_, CT_2_, CT_3_, CT_4_, and CT_5_ are 0.53, 0.683, 0.729, 0.812, and 0.939, respectively, indicating that as the distillation temperature increased, the number of alkyl substituents gradually decreased [27].

#### 3.1.2. Gas Chromatography Analysis

**Figure 4:**
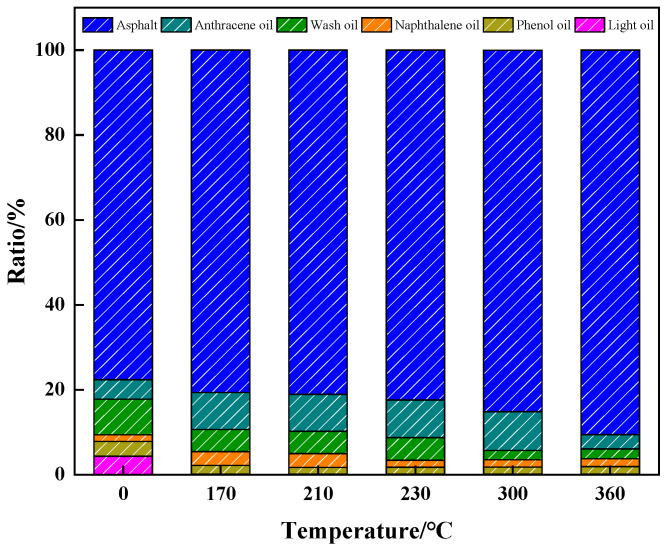
Coal tar fraction distribution.

Figure 4 shows the gas chromatography results regarding the distribution of coal tar fractions. As the distillation temperature rose, the percentage of pitch in the coal tar gradually increased. From the trends in each fraction, the proportions of light oil, phenol oil, naphthalene oil, and washed oil decreased and then increased, while the proportion of anthracene oil increased and then decreased. The lower boiling point fractions showed less change, while the higher boiling point fractions such as anthracene oil and pitch showed a significant change [28]. When the distillation temperature was higher than 170 °C, the light oil component within the coal tar was reduced by a large amount. The light oil mainly contained substances such as benzene and xylene, which had lower boiling points and were easily separated by distillation. Comparing the IR spectra of coal tar at a distillation temperature of 170 °C with the original coal tar, it was found that the peak areas of aliphatic hydrocarbons at 2920 cm^−1^ and 1450 cm^−1^ became smaller. When the distillation temperature was higher than 210 °C, most of the phenolic oil in coal tar was separated by distillation. The phenolic oil mainly contained m-p-cresol and other substances. The stretching vibration peak of -OH was at 3500 cm^−1^ in the combined infrared spectrum, it was found that the changes in functional groups were associated with the hydroxyl group as the temperature increased. When the distillation temperature was increased to 360 °C, the aromatic index I_ar_ of the coal tar was 0.3%.

#### 3.1.3. ^1^H-NMR

**Figure 5:**
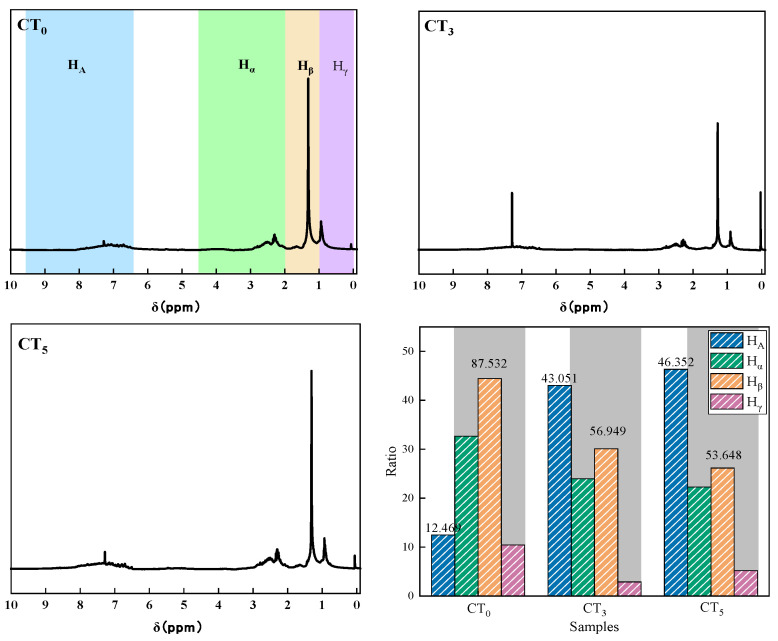
^1^H-NMR spectrum of coal tar.

Hydrogen NMR analysis was performed on the coal tar and the results are shown in Figure 5. Distilled coal tar contained less aromatic hydrogen, mostly aliphatic hydrocarbons, and more alpha and beta hydrogens, indicating a long fatty side chain. CT_5_ had more aromatic ring molecules, which indicates that increasing the distillation temperature was conducive to increasing the degree of aromatic ring condensation in coal tar. As the distillation temperature increased, the α-hydrogen, β-hydrogen, and γ-hydrogen contents of the coal tar decreased, indicating that the smaller molecules were separated by distillation and the longer fatty side chains were broken during the distillation process. This is consistent with the results of the IR analysis.

### 3.2. Mesophase Pitch Analysis

#### 3.2.1. Molecular Structure Analysis

**Figure 6:**
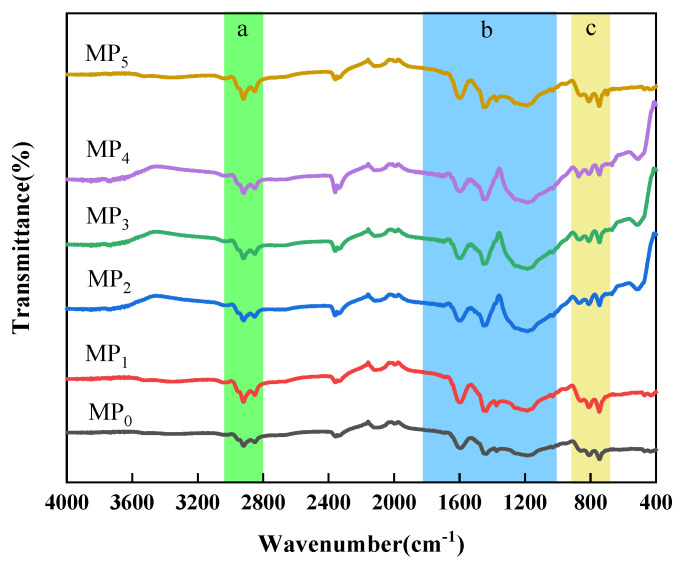
Infrared spectrum of pitch prepared by distillation of coal tar (a: 2800~3000 cm^−1^, b: 1000~1800 cm^−1^, c: 700~900 cm^−1^).

Figure 6 shows the infrared spectrum of pitch prepared from distilled coal tar. The mesophase pitch prepared is denoted as MP_0_, MP_1_, MP_2_, MP_3_, MP_4_, and MP_5_. The pitch has a large absorption peak at 1600 cm^−1^, which is the vibrational absorption peak of the C=C of the aromatic ring, indicating that the pitch has a structure of thick-ringed aromatics and benzene rings. A clear absorption peak at 2920 cm^−1^ is the C-H vibrational absorption peak of saturated methylene, and a methyl C-H vibrational absorption peak near 1450 cm^−1^ indicates that the pitch is rich in alkyl side chains and alkane structures. Symmetric and asymmetric vibrational absorption peaks in the C-H plane at 2920 cm^−1^, 2853 cm^−1^, and 1375 cm^−1^ indicate the presence of -CH_2_-CH_3_ functional groups in the coal tar. The presence of an absorption peak at 750 cm^−1^ but not at 720 cm^−1^ indicates the absence of longer alkyl side chains in the pitch.

**Figure 7:**
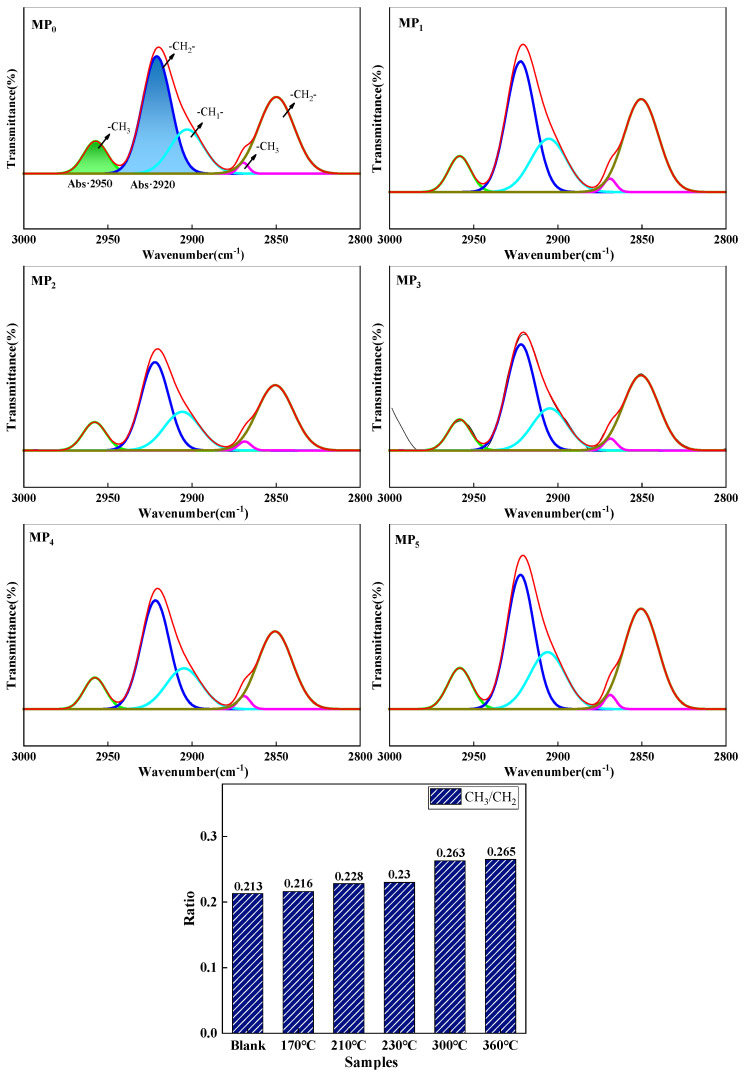
The peak fitting pattern of mesophase pitch at 2800~3000 cm^−1^.

Figure 7 shows the peak splitting fits for pitch at 2800 to 3000 cm^−1^. This region consists mainly of five functional groups: 2960 cm^−1^ and 2923 cm^−1^ are the telescopic vibrational peaks of asymmetric -CH_3_ and -CH_2_-; 2870 cm^−1^ and 2850 cm^−1^ are the telescopic vibrational peaks of symmetric -CH_3_ and -CH_2_-; and 2907 cm^−1^ is the telescopic vibrational peak of -CH-. This shows that increasing the distillation temperature reduced the number of alkyl substituents in the raw coal tar and facilitated the preparation of mesophase pitch with fewer alkyl substituents and shorter chain lengths. Combined with the analysis of the composition of the coal tar family, the HS was mainly composed of molecules with two to three rings, and with an increase in distillation temperature, such small molecules were separated by distillation. The HS might have had the function of acting as a solvent. It had the function of enhancing the fluidity of the system, facilitating the rearrangement of the lamellar structure within the intermediate phase, resulting in a wide range of mesophase structures. However, excessive HS content could have led to a lower mesophase content. Excess HS absorbed heat from the reaction system and was separated by distillation, which was unfavorable for the polymerization of the system. The HS benzene ring structure was relatively small and contained weak π-π bonds [29], as a result it was difficult for the molecules to align and stack. As the thermal polymerization reaction between the molecules occurred over an extended period, the molecular weight of the resulting aromatic molecules increased, and the molecules were arranged and stacked to form mesophase precursors.

**Figure 8:**
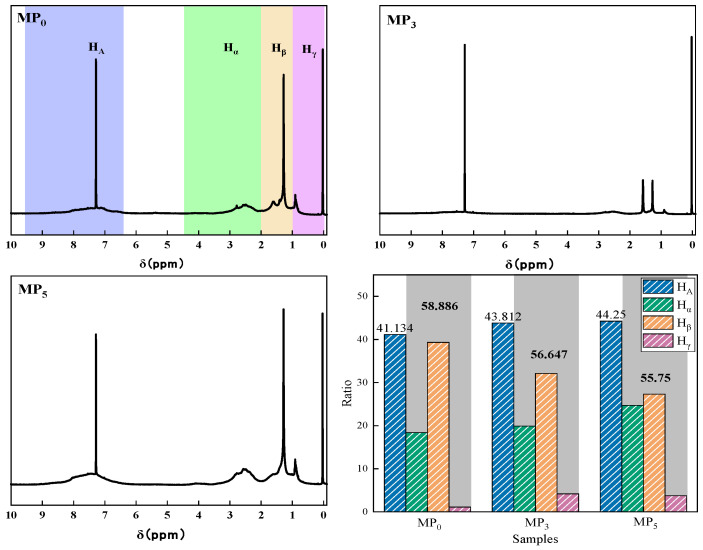
^1^H-NMR spectrum of mesophase pitch.

Figure 8 shows the ^1^H-NMR spectrum of the mesophase pitch. The chemical shifts of aromatic hydrogen (HA) were in the range of 6.5~9.5, the chemical shifts of aromatic-side-chain α hydrogen (Hα) were in the range of 2.0~4.5, the chemical shifts of aromatic-side-chain β hydrogen (Hβ) were in the range of 1.0~2.0, and the chemical shifts of aromatic-side-chain γ hydrogen (Hγ) were in the range of 0~1.0. MP_0_, MP_3_, and MP_5_ had larger peaks at this position, indicating that the mesophase pitch was dominated by polycyclic aromatic structures. As the distillation temperature increased, the β hydrogen content in the mesophase pitch decreased and the α hydrogen content increased. This indicates that increasing the distillation temperature, increasing the aromatic ring molecules and reducing the number of long chains and alkyl substituents, facilitated the preparation of mesophase pitch with a high degree of aromatic ring condensation, which is consistent with the IR analysis.

#### 3.2.2. Optical Structure Analysis

**Figure 9:**
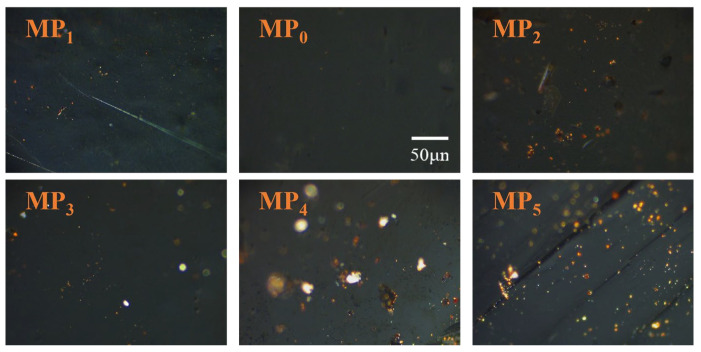
Polarization image of mesophase pitch.

Figure 9 shows a polarized picture of the mesophase pitch prepared by thermal polymerization of coal tar. The mesophase pitch prepared by thermal polymerization of coal tar mainly had a fine mosaic-type structure. The content of the prepared mesophase pitch increased as the distillation temperature of the raw material increased. During the distillation of coal tar, molecules with high thermal reactivity were gradually separated by distillation; the higher the distillation temperature, the less thermally reactive molecules the coal tar contains. The process of thermal polymerization of coal tar for the preparation of mesophase pitch mainly consisted of the orderly polymerization of aromatic molecules in a stacking reaction; the high thermal reactivity of coal tar made the polymerization reaction less active. Aromatic molecules could not effectively polymerize and stack; it was easy to form a mosaic structure of mesophase pitch when the mesophase content was low. CT_5_ had fewer lighter components and more aromatic molecules, so it was beneficial to reduce the lighter components in the raw material and increase the aromatic content of the raw material to increase the content of the mesophase pitch [30].

### 3.3. Effect of Reaction Conditions

The physical properties of the mesophase pitch are shown in Table 2. During the thermal polymerization of the coal tar, the composition and properties of the coal tar changed with the reaction temperature and reaction time. As the reaction time increased, the QI content of the mesophase pitch changed significantly, with a large increase before 12 h and a leveling off after 12 h. The HS and TI-QS also reached a stable state after 12 h. In the initial stages of the reaction, the reactively active pitch molecules cross-linked and polymerized to form mesophase molecules, resulting in significant changes in the QI, HS, and TI-QS. As the thermal polymerization reaction took place, the active large molecules in the raw material gradually decreased, the QI content increased, and the viscosity of the reaction system became larger [31]. When the cross-linking of molecules in the thermal polycondensation stacking could not occur easily, the polymerization reaction began to be slow. Therefore, it is presumed that 12 h was the time point at which the thermal polymerization reaction attained stability. It can be seen from the graph that the insoluble QI content showed a steep increase between 380 and 400 °C. The TI-QS content gradually decreased as the temperature increased, so that around 400 °C was the turning point of the reaction mutation.

#### 3.3.1. Effect of Reaction Time

**Figure 10:**
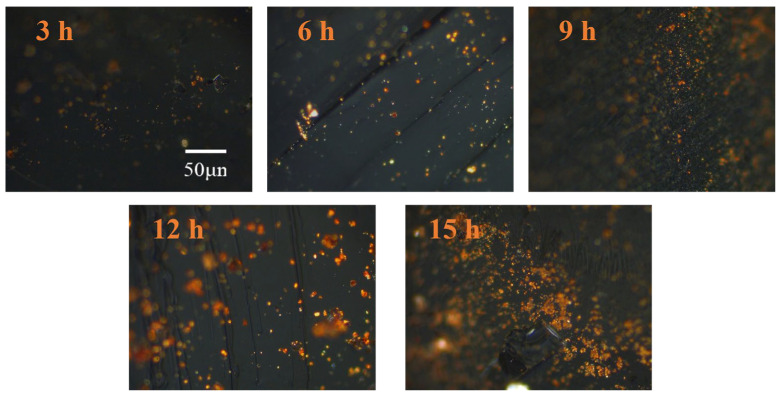
Polarized images of mesophase pitch prepared with different reaction times.

According to the traditional mechanism of the formation and development of middle-phase pitch, aromatic molecules in coal tar undergo thermal polymerization at high temperatures, resulting in the formation of planar aromatic macromolecules. Under thermal motion, these macromolecules begin to cross-link and stack into layers of a certain thickness. Due to surface energy effects, the layered structures gradually form middle-phase microspheres, which absorb the surrounding liquid and continue to grow, merging into larger spheres. As the reaction proceeds, the larger spheres break up to form mesophase pitch. As a result, the morphology of the mesophase changes as the reaction time increased, while extending the reaction time also produces more mesophases. From Figure 10 it can be seen that there is less mesophase, it is smaller in size, and it is not uniformly distributed. As the reaction time increased, the content of the mesophase significantly increased and became larger in size.

**Figure 11:**
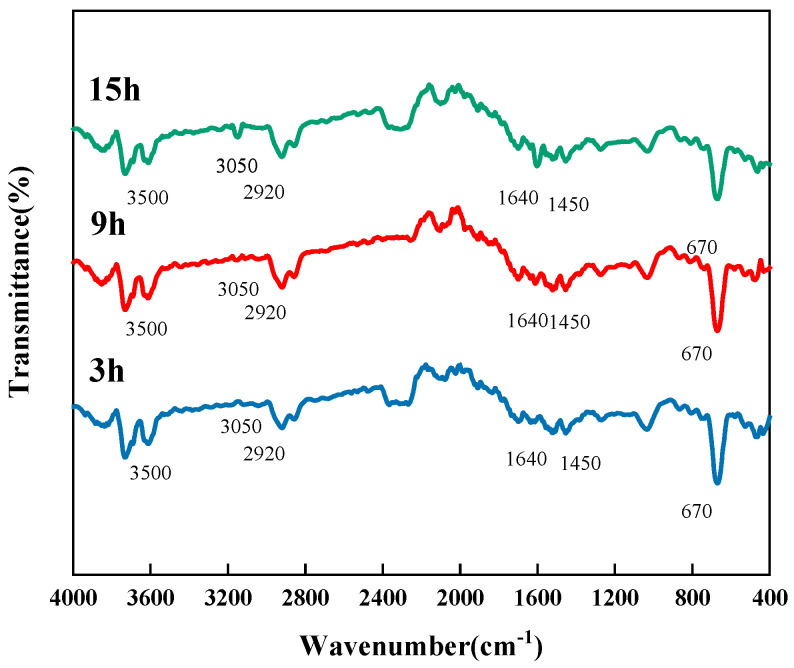
Infrared spectra of mesophase pitch prepared with different reaction times.

The infrared spectra of the mesophase pitch prepared with different reaction times are shown in Figure 11. In the range of aromatic hydrocarbons, a smaller absorption peak occurr near 3050 cm^−1^ and belongs to the vibrational absorption peak of C-H on the benzene ring. The absorption peak at 1600 cm^−1^ is the vibrational absorption peak of C=C of the aromatic ring. Near 670 cm^−1^ is the vibrational absorption peak of the carbon-hydrogen-substituted structure on the benzene ring. This indicates that the mesophase pitch prepared with all three reaction times had a structure of thick-ringed aromatics and benzene rings, with substitution reactions having occurred in the thick-ringed aromatics. In the region of aliphatic hydrocarbons, there is a distinct absorption peak at 2920 cm^−1^ for the C-H vibrational absorption peak of saturated methylene, the C-H vibrational absorption peak of methyl appears near 1450 cm^−1^. This indicates that all three mesophase pitches had alkyl side chains and alkane structures. There is a large absorption peak near 3430 cm^−1^, which is the stretching vibration peak of -OH. The IR profiles of the two are similar, with the mesophase pitch with a reaction time of 15 h having a more prominent absorption peak at 3050 cm^−1^ peak and vibrational absorption peak at 670 cm^−1^, indicating a higher degree of aromaticity. As the reaction time increased, the pitch produced more structures of thick-ringed aromatic and benzene rings.

**Figure 12:**
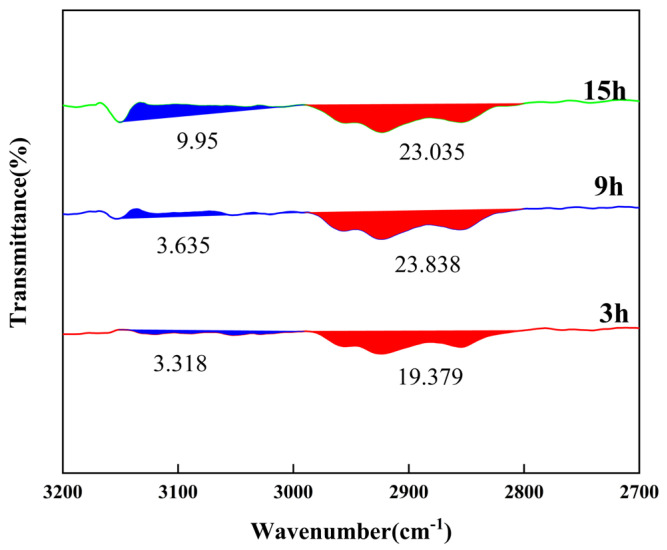
The peak fitting pattern of mesophase pitch at 2700~3200 cm^−1^.

To further compare the aromaticity of the two, the 3150 to 2900 cm^−1^ and 2900 to 2800 cm^−1^ profiles were integrated, as shown in Figure 12. The red area, indicating 2900 to 2800 cm^−1^, is denoted A_1_ and the blue area, indicating 3150 to 2900 cm^−1^, is denoted A_2_. According to the equation I_ar_ = A_2_/(A_1_ + A_2_), the aromatic index of the mesophase pitch with a reaction time of 3 h was found to be 14.62%. The aromatic index of the mesophase pitch with a reaction time of 9 h was 13.23% and the aromatic index of the mesophase pitch with a reaction time of 15 h was 30.17%. As the reaction time increased, the degree of aromatic condensation of the pitch increased significantly, indicating that molecules underwent a polymerization reaction, and the aromatic rings gradually became larger.

**Figure 13:**
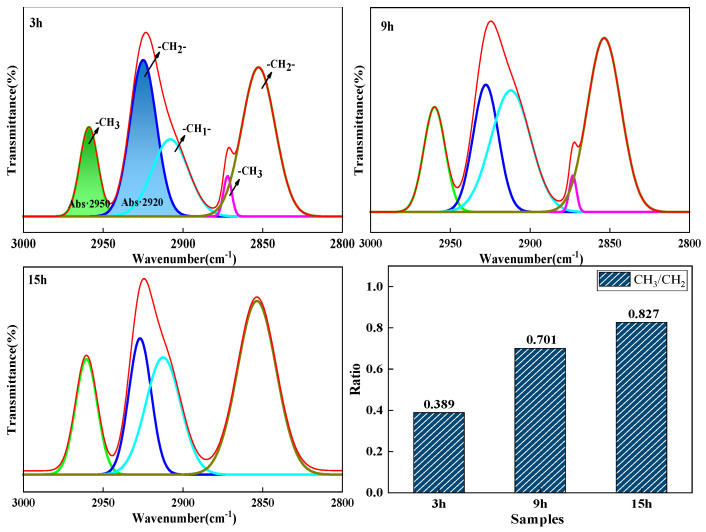
The peak fitting pattern of mesophase pitch at 2800~3000 cm^−1^.

Figure 13 shows the split-peak fit spectrum of pitch from 2800 to 3000 cm^−1^. The values of CH_3_/CH_2_ were 0.389 for a reaction time of 3 h, 0.701 for 9 h, and 0.827 for 15 h. As the reaction time increased, the value of CH_3_/CH_2_ gradually increased, the alkyl substituents of the pitch gradually decreased, and the chain lengths gradually became shorter. This suggests that the pitch underwent thermal polymerization, during which the molecular branched chains of the coal tar were likely to break, forming active sites that promoted polymerization between molecules.

#### 3.3.2. Effect of Reaction Temperature

**Figure 14:**
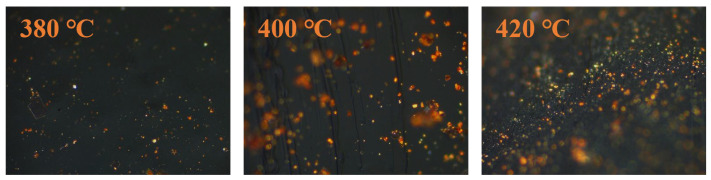
Polarized images of mesophase pitch prepared at different reaction temperatures.

Figure 14 shows that the mesophase pitch prepared at all three temperatures had a mosaic structure, with a and c being fine mosaics and b being a coarse mosaic. The coal tar feedstock obtained different forms of mesophases at different reaction temperatures, with thermal polycondensation reactions generally occurring at 350–500 °C. When the reaction temperature was too low the thermal polycondensation reaction failed to take place and no intermediate phase was produced, while at too high a temperature the reaction rate was too fast, and the intermediate phase produced was easily transformed into flaky coke and porous coke. When the reaction temperature was 380 °C, the viscosity of the reaction system was high, the cross-linking of the pitch molecules was limited, and the fusion of the mesophase spheres was also affected, thus no multi-phase system was produced. At 420 °C, the parallel arrangement of the mesophases also could not occur and the mesophases produced were generally of a fine mosaic-type structure. The amount of mesophase produced was low due to the slow reaction. At higher reaction temperatures, the reaction was more violent, the thermal polycondensation reaction activity was greater, the lighter components in the coal tar spilled out faster, and the smaller molecules received more energy, allowing them to condense more easily into aromatic macromolecules. It is easy to form a reaction system with high viscosity. The formed mesophase spheres were not easily fused with each other by the reaction system, so the resulting mesophase structure was similar to the low-temperature case, having a fine mosaic structure. As the reaction time increased, mesophase spheres were generated and grew. Due to the good reaction system, the mesophase spheres started to fuse with each other until a larger mesophase was formed.

**Figure 15:**
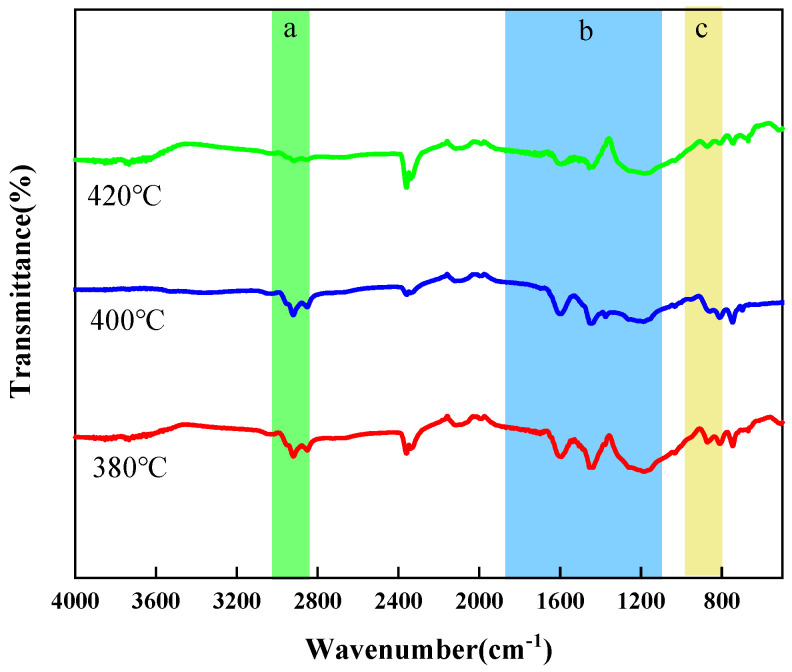
Infrared spectra of mesophase pitch prepared at different reaction temperatures (a: 2800~3000 cm^−1^, b: 1000~1800 cm^−1^, c: 700~900 cm^−1^).

As shown in Figure 15, the pitch has a large absorption peak at 1600 cm^−1^, which is the vibrational absorption peak of the C=C of the aromatic ring, indicating that the pitch has a structure of thick-ringed aromatics and benzene rings. There is a clear absorption peak at 2920 cm^−1^ for the C-H vibrational absorption peak of saturated methylene, and a C-H vibrational absorption peak for methylene near 1450 cm^−1^. This shows that the pitch has a more abundant alkyl side chain and alkane structure. Symmetric and asymmetric vibrational absorption peaks in the C-H plane at 2920 cm^−1^, 2853 cm^−1^, and 1375 cm^−1^ indicate the presence of -CH_2_-CH_3_ functional groups.

**Figure 16:**
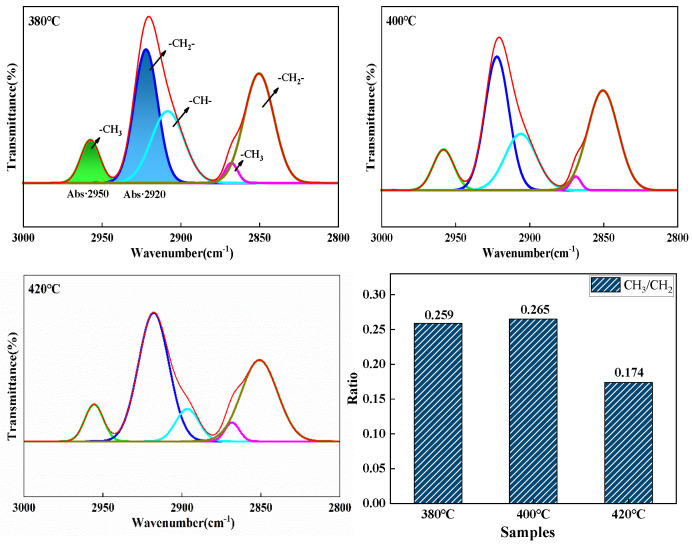
The peak fitting pattern of mesophase pitch at 2800~3000 cm^−1^.

Figure 16 shows the split-peak fit profiles for pitch from 2800 to 3000 cm^−1^. This region consists mainly of five functional groups. As the reaction temperature increases, the value of CH_3_/CH_2_ increases and then decreases, with the mesophase pitch prepared at 400 °C having the fewest alkyl substituents and the shortest chain length. This is because an appropriate increase in reaction temperature makes the thermal polymerization reaction proceed more thoroughly, making it easier for the molecular branched chains in the raw coal tar to break.

### 3.4. Mechanism

The aromatic molecules are bent and stacked by thermal stress to form aromatic spherical nuclei. The aromatic nuclei absorb aromatic molecules from the mother liquor to form mesophase spheres. Under suitable thermal polymerization conditions, the spheres absorb the aromatic molecules in the mother liquor, grow, collide, and dissolve into each other, and break up to form the bulk mesophase [32,33]. Mesophase pitch is mainly produced by thermal polycondensation reactions, where the thermal polycondensation process of the raw material can be divided into two stages. The first stage is the thermal decomposition reaction, in which aromatic or cycloalkane molecules undergo dehydrogenation at highly reactive sites and the breaking of long aliphatic chains, resulting in the production of a large number of reactive radicals and small molecules in the early stages of the reaction. The second stage is the thermal polycondensation reaction, in which the aromatic molecules undergo a free-radical polymerization reaction to form large molecules of thick-ringed aromatics. However, due to the very complex chemical composition and structure of the raw materials, their actual reaction process is also complex, so that thermal decomposition reactions and thermal polycondensation reactions may take place simultaneously.

**Figure 17:**
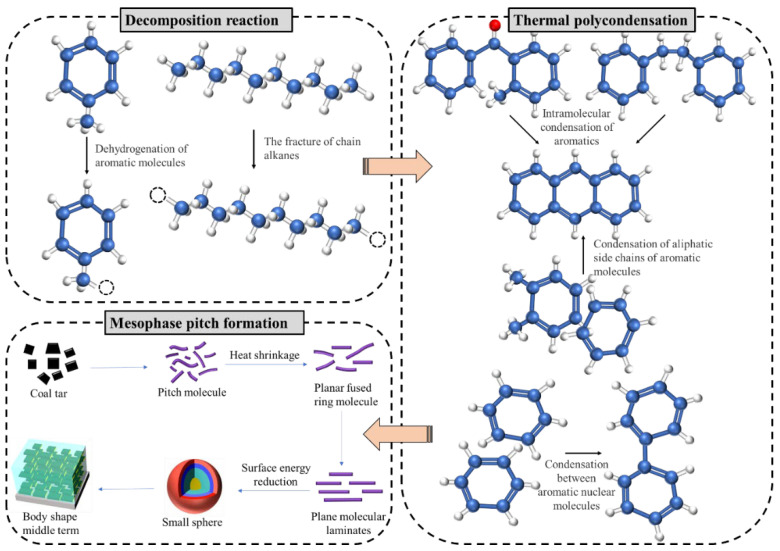
Formation mechanism of mesophase pitch.

The formation process of mesophase pitch is the result of a combination of chemical reactions and physical modulation. Figure 17 shows a flow diagram for the formation of mesophase pitch. In the nucleation phase of the mesophase structure, it is difficult for the aromatic molecules to align and stack because the structure of the aromatic molecules within the reaction system is relatively small and the aromatic molecules contain weak π-π bonds; i.e., the intermolecular forces between the aromatic molecules are relatively low. However, as the thermal polycondensation reaction between the aromatic molecules proceeds, the molecular weight of the resulting aromatic molecules increases, so this stage is dominated by the chemical reaction. In the early and middle stages of the formation of the mesophase structure, when large planar aromatic molecules are formed between the aromatic molecules by polymerization, the π-π bonds between the aromatic molecules are significantly stronger and the intermolecular forces allow the large planar aromatic molecules to be arranged in stacks, with certain rules. Moreover, the escape of small molecules from the system provides space for the contact of aromatic molecules. As the pile increases in size, the pile forms mesophase spheres under surface tension. At the same time, the small-molecule aromatic warp is still undergoing condensation reactions to form larger molecules and the mesophase spheres are growing. This stage therefore shows a combination of chemical reactions and physical modulation. Later in the formation of the mesophase structure, the growing mesophase spheres break up to form the bulk mesophase. Although the chemical reactions in the system are still going on, there are essentially no highly reactive aromatic molecules left; i.e., the chemical reactions are weak at this point [34]. Moreover, due to the increased viscosity of the system, the stacking of planar aromatic molecules is weaker, so this stage shows a weak combination of chemical action and physical modulation.

The formation of mesophase pitch is thus intricately linked to the thermal polycondensation of aromatic molecules. Consequently, the quantity and reactivity of aromatic molecules in the raw material dictate the mesophase content and the rate of mesophase pitch formation. Typically, the higher the concentration of aromatic molecules in the raw material and the greater their reactivity, the more readily mesophase pitch with a relatively high mesophase content can be formed. Regarding the aliphatic hydrocarbon structures, such as chain alkanes and cycloalkanes, present in the raw material, their primary function is to enhance the mobility of aromatic molecules, facilitating a more efficient mesophase formation process. Nevertheless, if the raw material contains an excessive amount of aliphatic hydrocarbon structures, it becomes challenging to form a mesophase structure. Therefore, the content of aliphatic hydrocarbon structures should be optimized to an appropriate level.

## 4. Conclusions

In this study, coal tar was subjected to distillation and treatment at temperatures of 170 °C, 210 °C, 210 °C, 230 °C, 300 °C, and 360 °C. The aim was to explore the impacts of varying coal-tar compositions and reaction conditions on mesophase pitch. The distilled coal tar and the synthesized mesophase pitch were analyzed using infrared spectroscopy, family-composition analysis, NMR, and optical structure analysis, and the following conclusions were obtained.

(1) The HS of coal tar decreased with increasing distillation temperature; the HI-TS and TI-QS increased with increasing distillation temperature. The value of QI was negligibly small. The coal tar had a thick cyclic aromatic and benzene ring-like structure with no longer alkyl side chains. As the distillation temperature increased, the content of lighter components decreased, the alkyl substituents in the coal tar gradually decreased, the chain length became shorter, and the aromatic condensation of the coal tar increased.

(2) The mesophase pitch was dominated by the structure of thick-ring aromatic hydrocarbons and benzene rings. Increasing the distillation temperature to appropriately reduce the number of alkyl substituents in the raw coal tar was conducive to the preparation of mesophase pitch with fewer alkyl substituents, shorter chain lengths, and a high degree of aromatic condensation. Coal tar at a distillation temperature of 360 °C, prepared at a reaction temperature of 400 °C and held for 12 h, resulted in a high degree of aromatic condensation of the mesophase pitch, with a high mesophase content and a predominantly mosaic-type structure.

(3) The formation of mesophase pitch was the result of a combination of chemical reactions and physical modulation. The chemical reactions mainly consisted of dehydrogenation of aromatic molecules and thermal polycondensation reactions. The molecular weight of the resulting aromatic molecules increased as the thermal polycondensation reaction between the aromatic molecules proceeded.

Increasing the distillation temperature leads to a higher degree of aromatic condensation in coal tar, favoring mesophase pitch formation, as Li [35] and Yang [21] have previously mentioned. This study also validates the conclusion proposed by Yu [36] that the molecular weight of mesophase pitch increases similarly after the thermal treatment of coal tar. However, discrepancies arise in the specific reaction conditions, such as the temperature and reaction time, which affect the molecular structure of the mesophase pitch. For instance, while some studies report efficient mesophase formation at lower temperatures (e.g., 300 °C) and shorter reaction times (e.g., 6 h), our findings suggest that a longer reaction time at 360 °C results in a more pronounced mesophase structure with a higher content of mesophase pitch. This discrepancy could be attributed to differences in raw coal tar composition or the specific distillation and reaction apparatus used in each study. Future research could explore these variables in greater detail to establish more precise correlations between reaction conditions and mesophase pitch properties, providing more accurate data support for the thermal processing of coal tar, enhancing the industrial foundation of this field, and minimizing resource waste.

## Figures and Tables

**Table 1:** Physical properties of coal tar.

Coal Tar	Composition/wt.%			
	HS	HI-TS	TI-QS	QI
CT_0_	92.3	6.89	0.81	-
CT_1_	85.26	13.83	0.91	-
CT_2_	73.34	24.15	2.51	-
CT_3_	58.66	39.51	4.83	-
CT_4_	39.16	50.46	10.38	-
CT_5_	17.83	58.37	23.57	0.23

**Table 2:** Physical properties of coal tar.

Performance	Temperature/°C	Time/h				
		3	6	9	12	15
HS	380	17.14	15.82	14.37	12.94	12.31
	400	16.21	15.53	14.75	13.22	12.63
	420	16.82	16.17	14.92	13.35	13.02
TI-QS	380	26.89	25.17	24.85	22.65	22.3
	400	26.33	25.02	24.23	21.94	21.67
	420	25.13	24.55	23.34	21.52	21.24
QI	380	5.43	11.76	24.34	36.71	38.33
	400	6.32	15.39	30.86	50.24	55.96
	420	7.19	18.62	34.14	60.67	65.74

## Data Availability

The data presented in this study are available on request from the corresponding author.

## References

[1] Zhao S.Q., Shao L.S. (2019). Analysis of the impact of a forecast of coal consumption on atmospheric environment. Fresenius Environ. Bull..

[2] Xu J.P., Wang F.J., Lv C.W., Xie H.P. (2018). Carbon emission reduction and reliable power supply equilibrium based daily scheduling towards hydro-thermal-wind generation system: A perspective from China. Energy Convers..

[3] Hishiyama Y., Inagaki M., Kimura S., Yamada S. (1974). Graphitization of carbon fibre/glassy carbon composites. Carbon.

[4] Fan B., Liu Y., He D., Bai J. (2017). Enhanced thermal conductivity for mesophase pitch-based carbon fiber/modified boron nitride/epoxy composites. Polymer.

[5] Li Z., Jiang Z., Ouyang T., Zhang Y., Ye C., Liu J. (2022). Unveiling the microcrystal structure evolution of carbon fibers induced by thermal soaking of mesophase pitch. J. Mater. Sci..

[6] Zhang L., Liu C., Jia Y., Mu Y., Yan Y., Huang P. (2024). Pyrolytic Modification of Heavy Coal Tar by Multi-Polymer Blending: Preparation of Ordered Carbonaceous Mesophase. Polymers.

[7] Wang M., Yang B., Yu T., Yu X., Rizwan M., Yuan X., Nie X., Zhou X. (2023). Research progress in the preparation of mesophase pitch from fluid catalytic cracking slurry. RSC Adv..

[8] Wang H., Xing Y., Xiao Z., Sun H., Wang G., Zhu M. (2022). Smart textiles for human-machine interface fabricated via a facile on-site vapor-phase polymerization. J. Mater. Chem. C.

[9] Zhang L., Deng F.M., Chen X.Z., Guo Z.H., Liu H.W., Xing X.T., Zhang Z.J. (2022). Microstructure graphitization evolution and multi-scale, multi-mechanism synergistic enhancement of ultra-high strength carbon-graphite materials. Diam. Relat. Mater..

[10] Yan L., Fang Y., Deng J., Zhu Y., Zhang Y., Cheng J., Zhao X. (2022). Preparation and Characterization of Mesocarbon Microbeads by the Co-Polycondensation of High-Temperature Coal Tar Pitch and Coal Pyrolytic Extracts. Materials.

[11] Dong Z.J., Sun B., Zhu H., Yuan G.M., Li B.L., Guo J.G., Li X.K., Cong Y., Zhang J. (2021). A review of aligned carbon nanotube arrays and carbon/carbon composites: Fabrication, thermal conduction properties and applications in thermal management. New Carbon Mater..

[12] Zhang L., Yan Y., Wang Y.S., Jia Y., Han Y.Z. (2022). Study on denitration performance of MnO_2_@CeO_2_ core-shell catalyst supported on nickel foam. Appl. Phys. A Mater..

[13] Banerjee C., Chandaliya V.K., Dash P.S. (2021). Recent advancement in coal tar pitch-based carbon fiber precursor development and fiber manufacturing process. J. Anal. Appl. Pyrolysis..

[14] Liu D., Li M., Qu F., Yu R., Lou B., Wu C., Niu J., Chang G. (2016). Investigation on Preparation of Mesophase Pitch by the Cocarbonization of Naphthenic Pitch and Polystyrene. Energy Fuels.

[15] Liang Z.W., Lu Y.G., Sun Z.L., Luo H. (2020). Polymerization kinetics and control of the components of a mesophase pitch. New Carbon Mater..

[16] Wang Y.L., He Z.G., Zhan L., Liu X. (2016). Coal tar pitch based carbon foam for thermal insulating material. Mater. Lett..

[17] Gabdulkhakov R.R., Rudko V.A., Pyagay I.N. (2022). Methods for modifying needle coke raw materials by introducing additives of various origin (review). Fuel.

[18] Song Q., Zou Y., Zhang P., Xu S., Yang Y., Bao J., Xue S., Liu J., Gao S., Lin L. (2025). Novel high-efficiency solid particle foam stabilizer: Effects of modified fly ash on foam properties and foam concrete. Cem. Concr. Compos..

[19] Liu Q., Li Y., Ming X., Zhao H., Sun Z., Li Z., Sun G. (2025). Optimizing concrete performance with polymer-cement networks: Enhanced flexural strength and crack resistance. Mater. Today Commun..

[20] Yang T., Liu B., Song Y., Ma Z.-K., Song H.-H., Liu Z.-J. (2020). Formation and Transformation Behavior of Mesophase from Three High Softening-Point Pitches. Carbon.

[21] Yang J.Y., Kim B.S., Park S.J., Rhee K.Y., Seo M.K. (2019). Preparation and characterization of mesophase formation of pyrolysis fuel oil-derived binder pitches for carbon composites. Compos. Part B Eng..

[22] Li M., Liu D., Du H., Li Q., Hou X., Ye J. (2015). Preparation of mesophase pitch by aromatics-rich distillate of naphthenic vacuum gas oil. Appl. Petrochem. Res..

[23] Zhao N., Liu D., Du H., Wang C.C., Wen F.S., Shi N. (2019). Investigation on Component Separation and Structure Characterization of Medium-Low Temperature Coal Tar. Appl. Sci..

[24] Dehghani M.H., Kamalian S., Shayeghi M., Yousefi M., Heidarinejad Z., Agarwal S., Gupta V.K. (2019). High-performance removal of diazinon pesticide from water using multi-walled carbon nanotubes. Microchem. J..

[25] Zhang L., Han Y., Shu H., Zhang L., Han Z., Yang X., Chen Y. (2023). Effect of bimetallic modification on blast furnace slag and its application in low-temperature selective catalytic reduction. J. Chem. Technol. Biotechnol..

[26] Xu R., Yang Z., Niu Y., Xu D., Wang J., Han J., Wang H. (2022). Removal of microplastics and attached heavy metals from secondary effluent of wastewater treatment plant using interpenetrating bipolar plate electrocoagulation. Sep. Purif. Technol..

[27] Niu Y., Yang Z., Wang J., Zhou Y., Wang H., Wu S., Xu R. (2022). Decomposition of perfluorooctanoic acid from wastewater using coating electrode: Efficiency, the anode characteristics and degradation mechanism. Sep. Purif. Technol..

[28] Niu Y., Yin Y., Xu R., Yang Z., Wang J., Xu D., Yuan Y., Han J., Wang H. (2022). Electrocatalytic oxidation of low concentration cefotaxime sodium wastewater using Ti/SnO_2_-RuO_2_ electrode: Feasibility analysis and degradation mechanism. Chemosphere.

[29] Song Q., Bao J., Xue S., Zhang P., Mu S. (2021). Collaborative disposal of multisource solid waste: Influence of an admixture on the properties, pore structure and durability of foam concrete. J. Mater. Res. Technol. JMRT.

[30] Song Q., Zhao H., Chang S., Yang L., Zou F., Shu X., Zhang P. (2020). Study on the catalytic pyrolysis of coal volatiles over hematite for the production of light tar. J. Anal. Appl. Pyrolysis.

[31] Song Q., Zhao H., Jia J., Yang L., Lv W., Bao J., Shu X., Gu Q., Zhang P. (2020). Pyrolysis of municipal solid waste with iron-based additives: A study on the kinetic, product distribution and catalytic mechanisms. J. Clean. Prod..

[32] Zhao H., Li Y., Song Q., Liu S., Ma L., Shu X. (2021). Catalytic reforming of volatiles from co-pyrolysis of lignite blended with corn straw over three iron ores: Effect of iron ore types on the product distribution, carbon-deposited iron ore reactivity and its mechanism. Fuel.

[33] Zhao H., Song Q., Liu S., Li Y., Wang X., Shu X. (2018). Study on catalytic co-pyrolysis of physical mixture/staged pyrolysis characteristics of lignite and straw over an catalytic beds of char and its mechanism. Energy Convers. Manag..

[34] Li Y., Zhao H., Sui X., Wang X., Ji H. (2021). Studies on individual pyrolysis and co-pyrolysis of peat-biomass blends: Thermal decomposition behavior, possible synergism, product characteristic evaluations and kinetics. Fuel.

[35] Li M., Zhang Y.D., Yu S.T., Xie C.X., Liu D., Liu S.W., Zhao R.Y., Bian B. (2018). Preparation and characterization of petroleum-based mesophase pitch by thermal condensation with in-process hydrogenation. RSC Adv..

[36] Yu Y., Lu Y., Cheng X., Han L., Yang C. (2022). Micro-kinetics of pitch polymerization with regards to molecular weight distribution. React. Chem. Eng..

